# Heterotopic gastric mucosa in the anus and rectum: first case report of endoscopic submucosal dissection and systematic review

**DOI:** 10.1093/gastro/gow006

**Published:** 2016-04-21

**Authors:** Federico Iacopini, Takuji Gotoda, Walter Elisei, Patrizia Rigato, Fabrizio Montagnese, Yutaka Saito, Guido Costamagna, Giampaolo Iacopini

**Affiliations:** ^1^Gastroenterology and Endoscopy Unit, Ospedale S. Giuseppe, Albano L., Rome, Italy,; ^2^Division of Gastroenterology and Hepatology, Nihon University School of Medicine, Tokyo, Japan,; ^3^Pathology Unit, Ospedale S. Giuseppe, Marino, Italy,; ^4^Endoscopy Division, National Cancer Center Hospital, Tokyo, Japan,; ^5^Digestive Endoscopy Unit, Policlinico Gemelli, Catholic University, Rome, Italy and; ^6^Gastroenterology Clinic, Rome, Italy

**Keywords:** heterotopic gastric mucosa, endoscopic submucosal dissection, review, rectum

## Abstract

**Background:** Heterotopic gastric mucosa (HGM) is the most reported epithelial heterotopia, but it is very rare in the rectum and anus.

**Methods:** The first case of an asymptomatic adult male with a large nonpolypoid HGM in the low rectum underwent complete resection by endoscopic submucosal dissection (ESD) is reported. The systematic review was based on a comprehensive search of MEDLINE, EMBASE and Google Scholar. Studies on humans were identified with the term ‘heterotopic gastric mucosa in the rectum and /or anus.’

**Results:** The search identified 79 citations, and 72 cases were evaluated comprising the present report. Congenital malformations were observed in 17 (24%) patients; rectal duplication accounted for most of the cases. The HGM was located in the anus and perineal rectum in 25 cases (41%) and low, middle and proximal pelvic rectum in 20 (33%), five (8%) and 11 cases (18%), respectively. Morphology was nonpolypoid in 37 cases (51%), polypoid in 26 cases (36%) and ulcerated in nine cases (13%). Specific anorectal symptoms were reported by 50 (69%) patients of the whole study population, and by 33 (97%) of 34 patients ≤ 18 years. Complications were observed in 23 cases (32%). The HGM was excised in 50 cases (83%). Endoscopic resection was performed in 17 cases (34%); resection was piecemeal in five of 12 lesions ≥15 mm, required argon plasma coagulation in two cases and was associated with residual tissue in two (17%). Intestinal metaplasia and an adenoma with low-grade dysplasia were described in three adults (4%).

**Discussion:** This systematic review shows that the HGM in the rectum and anus may be associated with specific rectal symptoms and serious complications, mainly in the pediatric population, and a risk of malignancy in adults. Its complete excision should be recommended, and the ESD can overcome the technical limits of conventional endoscopic snare resection and avoid unnecessary surgery.

## Introduction

Heterotopic mucosa refers to morphologically normal tissue displaced in a foreign anatomical site distinctly demarcated from the surrounding mucosa and entirely separated from its organ of origin [1]. Heterotopic gastric mucosa (HGM) is the most reported epithelial heterotopia and is classified either as congenital (heteroplasia) or acquired (metaplasia) when the result of an error in the positioning of endodermal stem cells during the organogenesis or the erroneous differentiation of pluripotent cells of a damaged epithelium, respectively [2,3]. Although a heteroplasia is suggested by oxyntic (fundic and body) mucosa and other congenital anomalies and/or heterotopias, the two entities are commonly known by the generic term ‘HGM.’

Reported ubiquitously, the HGM is predominantly observed in the gastrointestinal (GI) tract from the nose and glossopharynx region to the anus, biliary tract and pancreas [3–19]. Most cases are observed in the esophagus, duodenum and Meckel’s diverticulum. Its endoscopic prevalence ranges from 0.1% to 11% from the foregut and midgut [2,11,20], whereas it is rare in the hindgut [21]. The morphology and size of HGM are variable [22], and the differential diagnosis with neoplasms is established by biopsy sampling and histology [23].

This is the first systematic review of all cases in the literature and the first large nonpolypoid HGM resected in the low rectum by en bloc endoscopic submucosal dissection (ESD), a new technique that enables en bloc resections of superficial lesions regardless of their size and submucosal fibrosis. This study also defines the clinical spectrum of HGM in the rectum and anus and its risk of malignancy.

## Case report

**Figure 1.**
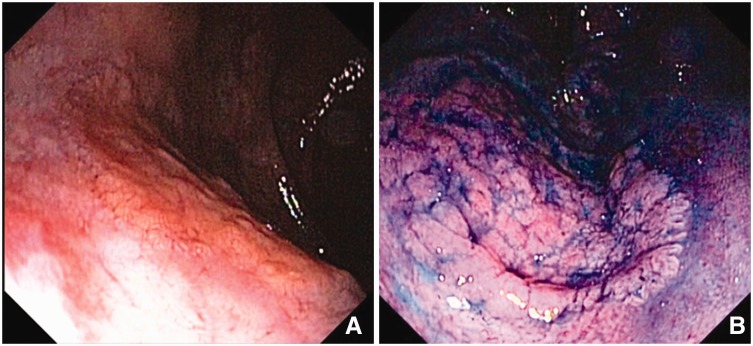
Heterotopic gastric mucosa in the low perineal rectum. (A) slightly elevated non-granular superficial lesion at white-light; (B) chromoendoscopy with indigo carmine in the retroflexed view.

An asymptomatic 63-year-old man was referred for colonoscopy due to a positive fecal blood test. Physical and digital rectal exams were normal. Laboratory studies were unremarkable. Colonoscopy revealed a laterally spreading, non-granular flat-type lesion (Paris classification 0-IIa), 25 x 25 mm in size, covering 25% of the circumference of the posterior wall of the rectum at 1 cm from the anal verge. Chromoendoscopy with 0.4% indigo carmine better delineated the lesion margins and showed a tubular pit pattern suggestive of a neoplasia (Figure 1).

**Figure 2.**
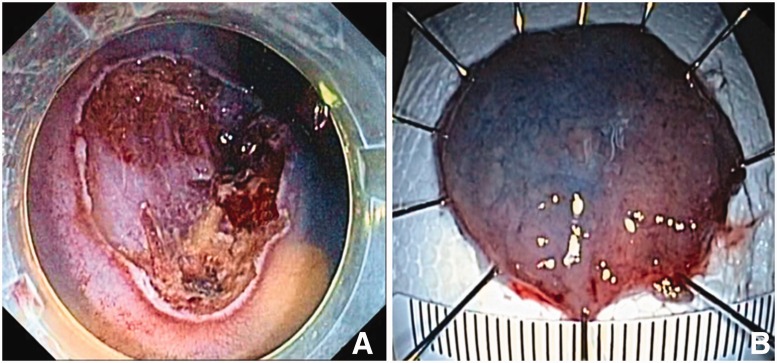
Endoscopic submucosal dissection: (A) resection site; (B) resected specimen 30 x 30 mm pin-oriented on foam.

Due to the endoscopic features suggestive of a superficial neoplasm, no biopsies were performed to avoid submucosal fibrosis, which would have increased the difficulty of resection. The patient provided written informed consent and underwent an ESD. A non-insulated knife (Dual-knife, Olympus) was used. Submucosal injection of a mixture of hydroxyethil starch, epinephrine (1:250 000) and indigo carmine showed a negative no-lifting sign, although mild submucosal fibrosis was observed during dissection. The lesion was resected en bloc with no adverse events (Figure 2).

**Figure 3.**
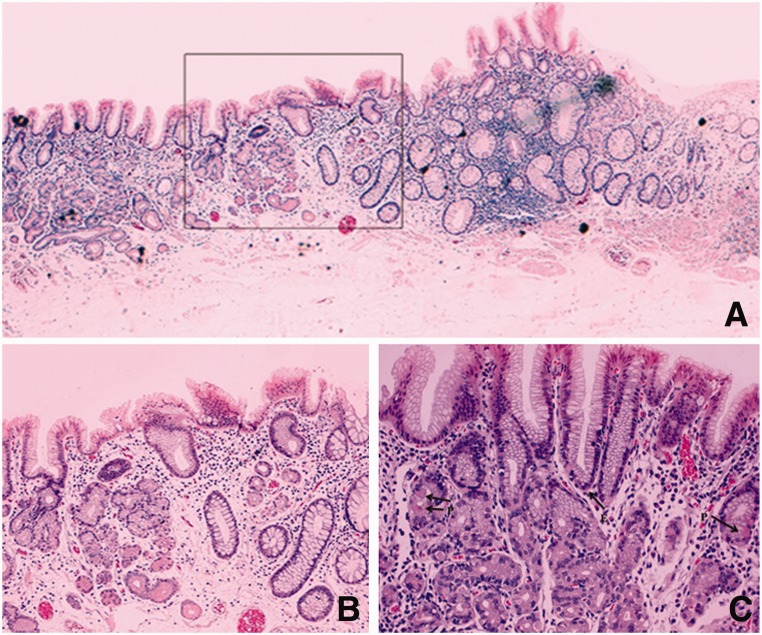
Histology (hematoxylin & eosin staining) showing heterotopic gastric mucosa of pyloric type in the rectum: (A) seriated section (magnification x5); (B) magnified view of border between gastric and rectal epithelium (boxed area in figure 3a) (magnification x10); C) predominant pyloric mucous glands with rare parietal (P) and endocrine (E) cells (magnification x20).

The specimen was stained with hematoxylin and eosin. Histology showed a flat lesion composed of predominant antral gastric mucosa with rare parietal and endocrine cells (Figure 3). No *Helicobacter pylori* organisms were detected with Giemsa staining. Endoscopic follow-up at 6 and 18 months confirmed the R0 resection.

## Methods

A search of the medical literature was conducted using MEDLINE, EMBASE and Google Scholar from 1939 (first description of rectal HGM) to September 2015. Studies on humans were identified with the term ‘heterotopic gastric mucosa’ (as a medical subject heading [MeSH] and a free-text term) and ‘heteroplas#’, ‘heterotop#’, ‘metaplas#’, ‘gastric mucosa’, ‘colon’, ‘rectum’ and ‘anus’ (as free-text terms). There were no language restrictions, and all potentially relevant papers were obtained and evaluated. The bibliographies of all studies were used to perform a recursive search of the literature. In case of multiple publications from the same authors, each case was identified by its demographic features, and redundancy was avoided.

The following data were extracted independently by two reviewers using predesigned forms with disagreements resolved by consensus: name of the first author, country of origin, year of publication; age and sex, size, morphology, location, histologic features of the HGM; symptoms leading to the diagnosis; duration of symptoms before diagnosis; presence of associated malformations/complications; treatment (conservative *vs* excision); technique of endoscopic resection (polypectomy, endoscopic mucosal resection, endoscopic submucosal dissection); technique of surgical resection (transanal, open, laparoscopic); follow-up period; recurrence of symptoms and recurrence rate after resection. The HGM morphology was reviewed according to the Paris classification [24] using both endoscopic images and text descriptions.

A two-sided *P* value < 0.05 was considered statistically significant. All of the calculations were performed with STATA software integration (StataCorp, Houston, Texas, USA).

## Results

Since the first description by Ewell and Jackson in 1939 [25], 78 cases of HGM in the rectum and anus have been reported, including the present one in the rectum. Six (8%) reports were excluded: two were redundant publications [26–28], and four were published in journals that were impossible to retrieve [29–32]. A total of 72 cases were evaluated (Table 1).

**Table 1.** Cases of heterotopic gastric mucosa in the rectum and anus sorted according to clinical category, location and size

Age/Sex	Malformation	Location	Morphology. (Paris)	Size (mm)	Complication	Mucosa type & Hp status	Treatment	Ref.
**Asymptomatic**								
68, F	No	–	0-IIa	10	No	Oxyntic, Hp neg	ER	[83]
53, M	No	–	0-IIa	25	No	Oxyntic, Hp neg	P-ER	[85]
46, M	No	–	0-Ip	30	No	Antral	ER	[70]
						+ adenoma		
69, M	No	Perineal	0-IIc	7	No	Oxyntic, Hp neg	ER	[23]
50, M	No	Perineal	0-Is	10	No	Oxyntic	–	[72]
60, M	No	Perineal	0-IIc	30	No	Mixed, Hp neg	Med	[73]
63, M	No	Perineal	0-IIb	30	No	Antral, Hp neg	ESD	Iacopini F, et al.
28, F	No	Low	0-I	–	No	Mixed	–	[64]
34, M	No	Low	0-Is	25	No	Oxyntic, Hp neg	ER	[65]
26, M	RD, (multiple)	Middle	0-Is	60	No	Oxyntic	Surgery	[38]
46, F	No	Prox	0-Ip	15	No	Oxyntic	Surgery	[3]
46, F	Rd	Prox	0-IIb	15	No	Oxyntic	No	[78]
51, M	No	Prox	0-II	30	No	Mixed + IM,	–	[54]
46, M	No	Prox	0-IIb	30	No	Cardiac + IM, Hp neg	No	[87]
**Nonspecific abdominal symptoms**								
55, M	No	Perineal	0-Is	5	No	Oxyntic, Hp neg	ER	[75]
36, F	No	Perineal	0-IIa-IIc	10	No	–	ER	[79]
34, F	No	Perineal	0-IIa	15	No	Oxyntic, Hp pos	–	[71]
65, F	No	Low	0-IIb	30	No	Oxyntic, Hp neg	ER	[88]
51, M	No	Low	0-IIa	40	No	Oxyntic, Hp neg	No	[86]
35, F	RD	Middle	0-IIb	10	No	Oxyntic, Hp pos	Med, Ablation	[60]
31, M	No	Middle	0-IIa	20	No	Oxyntic	Med	[63]
5, F	RD	Prox	Ulcer	–	Perforation	–	–	[43]
**Specific anal-rectal symptoms**								
3, F	no	–	ulcer	–	Fistula	–	–	[45]
24, M	scoliosis	–	0-I	–	No	Oxyntic	Surgery	[47]
10, M	RD, (multiple)	–	0-II	–	Fistula	–	Surgery	[56]
9, M	no	–	0-Is	–	No	–	Surgery	[62]
2, F	multiple	− *	0-Is	–	No	Oxyntic	Med	[55]
3, M	no	− *	Ulcer	–	Ulcer morph.	Oxyntic	Surgery	[90]
25, F	no	–	0-I	40	No	Oxyntic	–	[37]
15, F	no	− *	0-IIa	40	Ulcer (rectal)	Oxyntic	Med, Surgery	[58]
57, M	no	anus	0-IIa	8	Ulcer (anal)	–	Surgery	[89]
6, M	RD	Anus	Ulcer	10	Fistula	Oxyntic	Surgery	[67]
9, F	No	Anus	0-Ip	15	No	Oxyntic	ER	[61]
1.5, F	RD	Anus	Ulcer	20	Fistula	Oxyntic	Surgery	[48]
23, F	No	Perineal	Ulcer	–	Ulcer morph.	Oxyntic	Surgery	[33]
24, M	RD, scoliosis	Perineal	0-IIb	–	No	Oxyntic	Surgery	[42]
14, M	No	Perineal	Ulcer	–	Ulcer morph.	Oxyntic	Surgery	[10]
0.5, M	RD, digital	Perineal	0-IIa	–	No	Oxyntic	Surgery	[49]
4, M	No	Perineal	0-IIb	–	Fistula	Oxyntic	Surgery	[50]
21, M	No	Perineal	0-I	–	No	Oxyntic	Surgery	[12]
35, F	No	Perineal	0-Is	–	No	Oxyntic, Hp neg	Surgery	[75]
12, M	No	Perineal	0-Is	10	No	Mixed, Hp neg	ER	[84]
2, M	No	Perineal	0-IIc	15	Ulcer (rectal)	Mixed, Hp neg	Surgery	[68]
13, M	No	Perineal	Ulcer	20	Ulcer morph.	Oxyntic	Med, surgery	[26–28]
11, M	No	Perineal	0-IIb	25	No	Oxyntic	Surgery	[41]
6, M	No	Perineal	0-IIa-IIc	25	No	–	med, P-ER	[81]
17, M	No	Perineal	0-Is	30	Ulcer (rectal)	Oxyntic	Surgery	[52]
58, M	Rectal	Perineal	0-IIa	50	No	Oxyntic, Hp neg	No	[77]
6, M	No	Low	0-I	–	Ulcer (rectal)	Oxyntic	–	[25]
3, M	No	Low	0-I	–	No	Oxyntic	Surgery	[57]
58, F	No	Low	0-IIa	–	No	Oxyntic	–	[82]
19, F	Multiple	Low *	0-IIb	–	Perforation	Oxyntic	No	[39]
51, M	No	Low	0-IIb	1	No	Antral	Surgery	[3]
5, M	No	Low	0-Is	10	No	Oxyntic	Surgery	[36]
4.5, M	No	Low	0-Is	15	Ulcer (rectal)	Oxyntic	med, P-ER	[69]
1, F	No	Low	0-Is	25	No	Oxyntic	Surgery	[40]
36, F	No	Low	0-Is	25	No	Oxyntic, Hp pos	ER	[65]
16, M	No	Low	0-I	30	Ulcer (rectal)	Oxyntic	Surgery	[51]
2, F	No	Low	0-IIa	30	No	−, Hp neg	Med, surgery	[74]
48, F	RD	Low	0-IIb	30	No	Oxyntic, Hp neg	Surgery	[92]
4, F	RD	Low	0-II	35	No	Oxyntic	–	[34]
5, F	No	Low	0-IIa-IIc	40	No	Oxyntic	Med, P-ER	[80]
4, M	No	Low	0-IIa-IIc	50	Ulcer (rectal)	–	Med, surgery	[22]
22, M	No	Low	0-II	50	No	Oxyntic, Hp neg	–	[91]
7, M	No	Middle	0-Is	15	No	Oxyntic	ER	[3]
22, M	No	Middle	0-IIa-IIc	40	No	Oxyntic	Surgery	[46]
0, F	Multiple	Prox *	0-IIb	–	No	Mixed	–	[44]
20, M	No	Prox	Ulcer	15	Ulcer (rectal)	Oxyntic	Med	[53]
45, M	No	Prox	0-Ip	20	No	Mixed	P-ER	[35]
10, M	RD	Prox	0-IIa	20	Ulcer (rectal)	Oxyntic	Surgery	[66]
10, M	No	Prox	0-Is	30	Ulcer (anal)	Oxyntic	Surgery	[59]
47, F	No	Prox	0-IIa	30	No	Oxyntic, Hp pos	Med, ablation	[76]

ER, endoscopic resection; ESD, endoscopic submucosal dissection; F, Female; Hp, *Helicobacter pylori*; M, male; med, conservative treatment; P-ER, piecemeal endoscopic resection; RD, rectal duplication; *, multiple localization; IM, intestinal metaplasia

Cases by location were: 31 (43%) from Europe, 28 (39%) from North America, three (4%) each from the Middle East and India, three (4%) from Japan and South Korea and one from Colombia, New Zealand, Australia and South Africa, respectively.

Sixty-eight reports (94%) reports were single cases. The number of reports published from 2005 to 2015 (*n* = 27) was greater than those in previous decades (median = 9, range = 8–15) with a significantly higher rate of cases diagnosed as asymptomatic or with aspecific abdominal symptoms (14/27 *vs* 8/45; *P* = 0.004).

The median patient age was 22 years and ranged widely from the first day after birth to 69 years; 26 patients were ≤ 10 years; nine were 11–20 years, and 37 were >20 years. A total of 45 patients (63%) were males.

### Associated congenital malformations

Congenital malformations were observed in 17 patients (24%) (Table 1). Although intestinal duplications are extremely rare [93], with rectal duplications being more so (1–8% of all cases) [94,95], rectal duplication was the most prevalent malformation being observed in 12 cases (71%). Other malformations of the GI and genitourinary tracts and skeletal system occurred in eight cases and were generally multiple: anal stenosis and sacrococcygeal defect (‘Currarino triad’) [38,56]; digital anomalies (clino- and syndactylia) [49]; myelomeningocele, malplaced (anterior) and imperforate anus [55]; enterocloacal fistula, multiple genitourinary abnormalities, malrotation and/or partial atresia of the colon [39,44]; spina bifida, pectus excavatum and bicornate uterus [39]; scoliosis [42,47] and Meckel’s diverticulum [38,55]. Rectal duplication and other malformations were combined in four cases [38,42,49,56]. The association of HGM in the rectum and anus—either with rectal duplication and digital anomalies [49] or an anal stenosis and sacrococcygeal defect [38,56]—are considered hereditary syndromes. Salivary [3] and pancreatic heterotopias [3,44] were observed in three cases.

### Pathologic features

#### Localization

HGM has generally been described as a solitary lesion electively localized at the right posterior wall of the rectum. Multifocal localization was reported in five cases (7%) [39,44,55,58,90]. Specifically described in 61 cases (85%), the localization of HGM can be stratified in four segments of 3 cm in length (Table 1): anus and perineal rectum in 25 cases (41%) (4 and 21 cases, respectively), low, middle and proximal pelvic rectum in 20 (33%), five (8%) and 11 (18%) cases, respectively.

#### Morphology

The HGM morphology was redefined according to the Paris classification [24] in all cases. It was nonpolypoid in 37 cases (51%), polypoid in 26 (36%) and ulcerated in 9 (13%) (Table 1). In 52 cases (72%), the morphology was better characterized as the following types: nonpolypoid slightly elevated (type 0-IIa) in 13 cases (25%) including the present case, nonpolypoid flat (type 0-IIb) in 12 cases (23%), nonpolypoid slightly elevated with a pseudo-depression (type 0-IIa-IIc) in five cases (10%), nonpolypoid depressed (type 0-IIc) in three cases (6%), polypoid sessile (type 0-Is) in 15 cases(29%) and polypoid pedunculated (type 0-Ip) in four cases (8%). The median size was 25 mm and ranged between 1 mm and 60 mm [3,38].

HGM-related complications were observed in 23 cases (32%): ulcer of the adjacent rectal and anal mucosa in 11 cases (15%), ulcerated morphology *per se* in nine cases (13%), fistula in five cases (7%) (rectovesical in two [45,50]; trans-sphyncteric in two [56,67], anocutaneous in one [48]) and colon perforation in two cases (3%) [39,43].

A large polypoid neoplasm [38] and invasive cancer [3] were observed close to the HGM site in a 26-year old asymptomatic male and a 51-year old adult male with hematochezia.

#### Histology

The histologic type of the HGM has been reported in 63 cases (88%) (Table 1). The oxyntic mucosa was the most prevalent, being observed in 52 cases (83%); mixed oxyntic and antral mucosa was found in seven cases (11%), antral mucosa in three cases (5%) and cardiac mucosa in one case (2%). Enterochromaffin-like (ECL) cells were described in two cases (3%) [26,68].

Advanced histologic changes, i.e. intestinal metaplasia and a pyloric adenoma with low-grade dysplasia, were observed in two cases (3%) [54,87] and one case (2%) [70], respectively. These three cases of HGM with preneoplastic and neoplastic changes (5%) were incidentally diagnosed in asymptomatic adults (Table 2), which represented 14% of the 21 adults ≥ 45 years, and 21% of the 14 asymptomatic cases.

**Table 2.** Demographic and heterotopic gastric mucosa features according to the symptom categories. Statistical analysis performed by the chi-square test (*P* value refers to all three groups).

	Symptom categories			P=
	Asymptomatic	Nonspecific (abdominal)	Specific (rectal)	
	(*n* = 14)	(*n* = 8)	(*n = *50)	
Males	10 (71%)	3 (38%)	32 (64%)	0.272
Age at diagnosis (yrs), median (range)	48 (26–69)	36 (5–65)	11 (0–58)	<0.0001
Age at diagnosis (yrs), *n* (%)				<0.0001
≤ 10	0	1 (13%)	25 (50%)	
11–18	0	0	8 (16%)	
>18	14 (100%)	7 (88%)	17 (30%)	
Localization: A + Perineal / L / M + Prox	4 / 2 / 5 ∗	3 / 2 / 3	18 / 16 / 8 ∗	0.392
Morphology: NP / P / U	8 / 6 / 0	6 / 1 / 1	23 / 19 / 9	0.257
Size, mm, median (range)	25 (7–60)	15 (5–40)	25 (1–50)	0.444
Histology: oxyntic /non-oxyntic	11 (85%) / 3	6 (100%) / 0 ∗	42 (98%) / 1 ∗	0.031
Histology: IM / dysplasia	3 (21%)	0	0	0.002
Complications	0	1 (13%)	22 (44%)	0.004

A + P, anus and perineal rectum; IM, intestinal metaplasia; L, low rectum; M + Prox, middle and proximal rectum; NP, nonpolypoid; P, polypoid; U, ulcerated; ∗ incomplete data; yrs, years

Acid secretion of the oxyntic HGM has been demonstrated in response to pentagastrin [46] or histamine by pH probes [26,35] and by Congo red vital staining [26]. Active acid secretion has been indirectly demonstrated in five cases in whom an H2-receptor antagonist or proton pump inhibitor (PPI) treatment course achieved healing of the ulcerated HGM itself or the associated ulcer [22,26,53,58,69]. A technetium-99m pertechnetate scan performed in nine children [22,49,55,66–69,74,81] showed rectal radionuclide accumulation in only three cases (33%) [69,74,81].

Since its discovery in 1982, the *Helicobacter pylori* status evaluated by Giemsa or Warthin-Starry stains was positive in four (19%) of 21 cases. In one case, *Helicobacter pylori* was present at the HGM in the rectum but not in the stomach [65].

### Clinical features

Symptoms and signs leading to the diagnosis of HGM in the anus and rectum were divided in three categories based on their assumed relationship with the HGM: (i) absent (incidental diagnosis), (ii) nonspecific abdominal, not rectal/anal and (iii) specific rectal-anal.

Incidental diagnoses of HGM in the rectum and anus occurred mainly in the last decade due to the expanded use of colonoscopy for CRC screening, irritable bowel syndrome and dyspepsia. Nonspecific abdominal symptoms—not anorectal—were bowel habit changes, bloating, discomfort in the lower abdominal quadrants and cramping pain. Hematochezia, described as acute or chronic recurrent bright red blood passage streaked on the stool, pants and toilet paper was the most frequent specific rectal-anal symptom. Life-threatening bleeding has been described in two cases [49,58]. The other specific symptoms were anal pain, tenesmus, burning or pruritus ani. Symptom duration before diagnosis was extremely variable from days and months to years.

Patients were asymptomatic in 14 cases (19%). In this group, 5 patients had a positive fecal occult blood test and/or iron-deficiency anemia. Nonspecific abdominal symptoms were reported by eight patients (11%). Specific anorectal symptoms were reported by 50 patients (69%), in association with nonspecific symptoms in 12 cases (Table 1).

Compared with asymptomatic patients and patients with nonspecific abdominal symptoms, those with specific anorectal symptoms were significantly younger, more frequently of pediatric age (≤18 years) and had a significantly higher prevalence of HGM-related complications (ulcer, fistula, bowel perforation as described in the Morphology paragraph) (Table 2). Only one five-year-old girl with rectal HGM reported nonspecific abdominal pain and a perforation at the rectosigmoid junction [43]. Finally, specific anorectal symptoms were significantly associated with a higher prevalence of gastric oxyntic mucosa.

## Treatment

Data on treatment are available for 60 cases (83%). The primary approach consisted of excision in 42 cases (70%), and conservative treatment with H2 receptor antagonist, PPI, bismuth subsalicylate and antibiotics for *H. pylori* in 13 cases (22%), most of whom (*n* = 8) were children with specific symptoms (Table 1). Five (8%) patients underwent an observational follow-up, and three refused resection [39,78,86].

Conservative treatment was effective both in symptom control and ulcer healing in all cases, but resection or ablation was performed in eight (62%) cases after a three-month period (range = 1–8), and in four patients due to early symptom recurrence at treatment withdrawal [58,74,76,80].

Irrespectively of symptoms and pathology, the definitive treatment of the HGM was its resection in 50 cases (83%) and ablation in two cases (3%). Surgery was the exclusive approach up to the early 1990s when endoscopic resection becomes preferred whenever feasible. Overall, HGM excision was performed by surgery in 33 cases (66%) and endoscopically in 17 cases (34%). Surgery, most often transanal, has been the preferred approach for HGMs with rectal duplication, ulcerated morphology and/or complication by fistula and perforation, not amenable of endoscopic resection. Only one nonpolypoid HGM in a small rectal duplication was treated by endoscopic ablation [60]. Moreover, HGM lesions resected surgically were larger (median 25 mm, range 10–60 mm) than those underwent endoscopic resection (median 20 mm, range 5–40 mm).

Complete endoscopic snare resection (polypectomy and endoscopic mucosal resection, EMR) was achieved in multiple pieces (piecemeal resection) in five (42%) of 12 lesions ≥ 15 mm [35,69,80,81,85]. Ablation at the resection site with argon plasma coagulation was performed in two cases (17%) due to suspicious residual tissue [79,80]. A residual HGM area was detected during the follow-up after resection in two (17%) of 12 cases [69,79]. The present case is the first to report successful en bloc endoscopic resection of a large non-polypoid, slightly-elevated HGM by ESD.

After the HGM excision, no symptoms recurred in any cases within a median follow-up of 22 months (range 2–84).

## Conclusions

The present systematic review of all cases of HGM in the anus and rectum introduces enables detection, for the first time, a clinico-pathologic classification that may be helpful for identifying both the clinical relevance and prognosis.

Specific symptoms and serious complications indicate that HGM of the rectum and anus should be considered in the differential diagnosis of the rectal syndrome in young patients.

The three cases of intestinal metaplasia and pyloric adenoma in the rectal HGM identified in the present review, [54,70,87] cancers from HGM in animals [96], and rare cancers from HGM in the esophagus [97,98], small bowel, gallbladder and colon in humans [97,99,100], suggest that HGM has a risk of malignancy. The present three cases of HGM with intestinal metaplasia and adenoma were incidentally diagnosed in asymptomatic adults during screening colonoscopy, and represent the 14% of adults ≥45 years and the 21% of all asymptomatic cases. This result indicates that congenital long-standing HGM in the rectum and anus may have a relevant risk of preneoplastic and neoplastic change and that this condition should be followed endoscopically and/or excised whenever possibile. This observation needs to be confirmed in asymptomatic HGM cases that would likely be identified in adults by colorectal screening programs.

Conservative treatment has only been temporarily effective and only used before definitive HGM excision. The technical limits of conventional endoscopic snare resection played an important role in the choice between surgery and endoscopy and resulted in the decision for surgery. Actually, conventional endoscopic snare resection of nonpolypoid flat and depressed lesions is difficult due to (i) the slipperiness of the snare over the lesion and (ii) the presence of submucosal fibrosis causing a positive no-lifting sign. The submucosal fibrosis observed during dissection in the present case can be a common finding in HGM due to the associated chronic gastritis and ulcerative complications. In this context, the ESD is an effective, minimally invasive approach that overcome the limits of conventional snare resection (polypectomy and EMR), achieves a complete resection regardless of the lesion size and avoids unnecessary surgery [101]. Although more difficult and risky than conventional endoscopic resection, the rectal location of the HGM may be favorable for the adoption of ESD since a extraperitoneal perforation is less clinically relevant than in the periotneal colonic perforation [102]. Regardless of future data on the malignancy risk of HGM, ESD may have a positive cost-benefit balance in long-term in relation to an accurate pathologic evaluation; negligible rate of residual tissue/recurrence; no need for follow-up and reinterventions [101].
